# Photo-induced ligand substitution of Cr(CO)_6_ in 1-pentanol probed by time resolved X-ray absorption spectroscopy†

**DOI:** 10.1039/d1cp05834g

**Published:** 2022-06-07

**Authors:** Eric J. Mascarenhas, Mattis Fondell, Robby Büchner, Sebastian Eckert, Vinícius Vaz da Cruz, Alexander Föhlisch

**Affiliations:** Universität Potsdam, Institut für Physik und Astronomie 14476 Potsdam Germany eric.mascarenhas@helmholtz-berlin.de; Helmholtz-Zentrum Berlin für Materialien und Energie GmbH, Institute for Methods and Instrumentation for Synchrotron Radiation Research 12489 Berlin Germany

## Abstract

Cr(CO)_6_ was investigated by X-ray absorption spectroscopy. The spectral signature at the metal edge provides information about the back-bonding of the metal in this class of complexes. Among the processes it participates in is ligand substitution in which a carbonyl ligand is ejected through excitation to a metal to ligand charge transfer (MLCT) band. The unsaturated carbonyl Cr(CO)_5_ is stabilized by solution media in square pyramidal geometry and further reacts with the solvent. Multi-site-specific probing after photoexcitation was used to investigate the ligand substitution photoreaction process which is a common first step in catalytic processes involving metal carbonyls. The data were analysed with the aid of TD-DFT computations for different models of photoproducts and signatures for ligand rearrangement after substitution were found. The rearrangement was found to occur in about 790 ps in agreement with former studies of the photoreaction.

## Abstract 2

The photo-induced ligand substitution reaction of Cr(CO)_6_ in 1-pentanol was investigated using time-resolved X-ray absorption spectroscopy. The results point to ligand rearrangement after photoexcitation.

## Graphical abstract

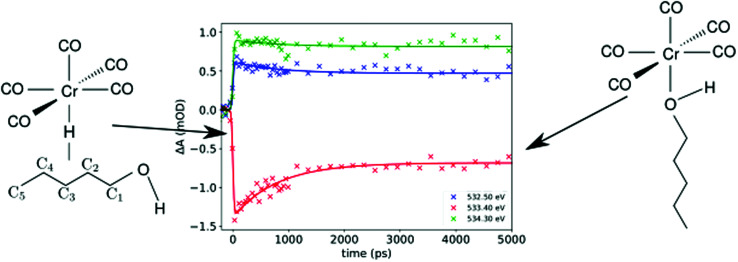

## 1 Introduction

To reach an electronic closed shell – the noble gas configuration – transition metal (TM) atoms coordinate to a number of ligands allowing for a saturated closed shell complex. This principle is captured by the 18 electron rule formulated by Langmuir.^1^ Following this rule, coordination with carbonyl ligands with 3d TMs forms stable, saturated complexes, namely, for nickel as Ni(CO)_4_, iron as Fe(CO)_5_, and chromium as Cr(CO)_6_. In addition to the basic 18 electron rule, specific factors, such as electronegativity, bonding character, symmetry and conformation, contribute to the stability of transition metal complexes. These aspects can be rationalized within the Dewar–Chatt–Duncanson model^2^ based on donation and back-donation between orthogonal ligand states and the TM d-states. This model explains the prominent role of CO in the spectrochemical series based on its ability to bind to the metal *via* both σ-donation and π-back-donation channels. Although general in nature, the compensating interaction of the σ and π orbitals for TM complexes is only stringent as long as σ and π derived orbitals of the TM complex are orthogonal. This condition is fulfilled in the octahedral Cr(CO)_6_ complex, but significantly altered by the additional strong mixing due to the lower symmetry of the tetrahedral Ni(CO)_4_ and the trigonal bi-pyramidal Fe(CO)_5_ complexes.

**Fig. 1:**
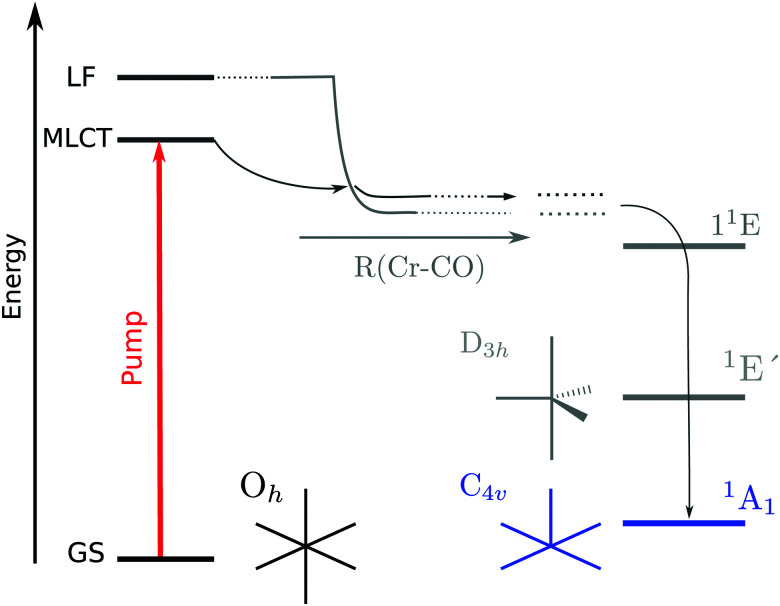
Photo-induced dissociation pathway in Cr(CO)_6_. After pumping, the system falls into a dissociative potential in which the square pyramidal Cr(CO)_5_ is formed. Adapted from Trushin *et al.*^3^

The higher symmetry of Cr(CO)_6_ grants a great simplification on its photochemistry, due to the orthogonality of the σ- and π-derived electronic orbitals. Fig. 1 illustrates the established mechanism of ligand dissociation in Cr(CO)_6_ following ultra-violet excitation. The system rapidly reaches the square-pyramidal unsaturated species Cr(CO)_5_ in the ^1^A_1_ state.^4^ It is important to emphasize that both the starting point (*O*_h_ Cr(CO)_6_) and end point (*C*_4v_ Cr(CO)_5_) of ligand dissociation are in configurations where the π and σ bonding channels are orthogonal, fitting into the Dewar–Chatt–Duncanson model. Although in the gas phase further dissociation occurs, in the solution phase the square-pyramidal species is stabilized. This provides an unambiguous starting point from which the subsequent ligand attachment reaction can be studied.

**Fig. 2:**
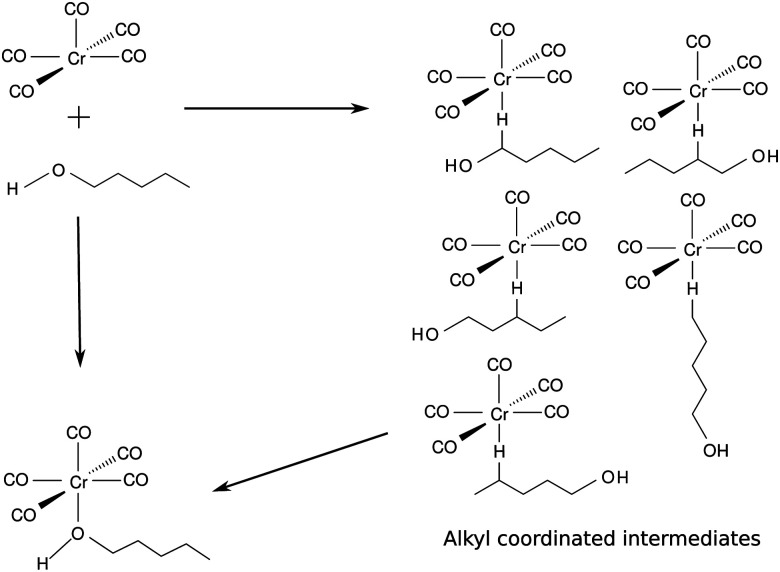
Dynamics of ligand attachment to the square pyramidal intermediate, following ejection of a CO ligand of Cr(CO)_6_ in 1-pentanol.

In solution, starting from the well defined pentacoordinated Cr(CO)_5_ species, we can investigate how the ligand attachment takes place. The photosubstitution reaction of Cr(CO)_6_ was shown to take place in many solvents.^5–8^ However, an interesting process takes place in the case of long chain alcohols. Early on Xie and Simon^9^ noticed that both alkyl and hydroxyl sites take part in coordination. In 1-pentanol the unsaturated Cr(CO)_5_ can bind to the incoming solvent through any of the five carbons in the alkyl chain or through the hydroxyl end of the incoming ligand.^9,10^ See Fig. 2 for a schematic depiction of this process. The free electron pair in the oxygen atom of the alcohol grants the condition of a strong σ ligand while the interaction with the alkyl moieties is characterized by a weak σ interaction^11^ involving the carbon and hydrogen atoms of the alkyl side of the alcohol. At first, the binding coordination site is random and the alkyl coordinated species changes its coordination site until the hydroxyl end of the solvent is able to coordinate to the metal, acting as a trap for the photoproduct.^12,13^ This linkage isomerism was observed for the class of metal carbonyls in a series of long chain ligands.^14,15^

In this work, we investigate the photo-induced ligand substitution reaction of Cr(CO)_6_ in 1-pentanol using multi-edge soft X-ray absorption spectroscopy. The steady state spectra of the complex at the Cr L_3,2_-, and O K-edges will be presented along with time-resolved X-ray absorption spectroscopy to monitor the photo-induced ligand substitution reaction of Cr(CO)_6_ in a solution of 1-pentanol.

## 2 Experimental

Cr(CO)_6_ was purchased from Santa Cruz Biotechnology and 1-pentanol from Carl Roth. Both chemicals were used without further purification. Cr(CO)_6_ was dissolved in pure 1-pentanol to a concentration of 14 mM.

With the nmTransmission NEXAFS end station, the liquid sample is transported into the vacuum chamber through two nozzles. The constant flow of new sample at the X-ray focus spot avoids radiation induced sample damage. The two jets collide forming a leaf where the thickness can be adjusted by varying the flow rate of the HPLC pump. In our experiment, a flow rate of 2.6 mL min^−1^ and 46 μm nozzles were used.

The experiment was performed at the UE52-SGM^16^ beamline at BESSY II. The static O K-edge spectrum was acquired with 250 meV bandwidth of the incoming radiation with a gallium arsenide photodiode, while the transient spectrum was recorded with 120 meV bandwidth with a silicon avalanche photodiode capped with a 200 nm Al film. For the acquisition of the final static spectrum at the O K-edge, the pure solvent spectrum was subtracted from the acquired signal due to the high absorption observed as background. The photoexcitation was induced by the use of the 4th harmonic (258 nm) of a fiber laser system with a fundamental wavelength of 1030 nm. The dynamics at the O K-edge was measured with this laser system focused on a (60 × 65) μm^2^ FWHM spot. For the dynamic measurement at the Cr L_3,2_-edge, the laser spot size was (80 × 80) μm^2^ FWHM. Comparing with tabulated values of attenuation^17^ in the solvent, the sample thickness was estimated to be in the range of 1–2 μm throughout the experiments. Further details about the experimental setup have been described by Fondell *et al.*^18^ Supplemental measurements were performed with the EDAX^19^ endstation at the UE49-SGM^20^ beamline at BESSY II.

**Fig. 3:**
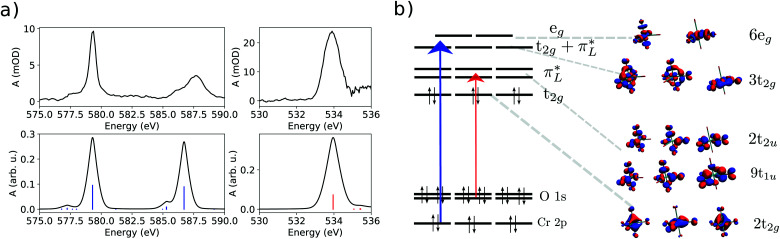
(a) Experimental ground state (static) NEXAFS spectrum for the Cr L_3,2_-edge (left) and O K-edge (right). Below, the computation is shown; (b) simplified orbital diagram with molecular orbitals computed for Cr(CO)_6_.

**Fig. 4:**
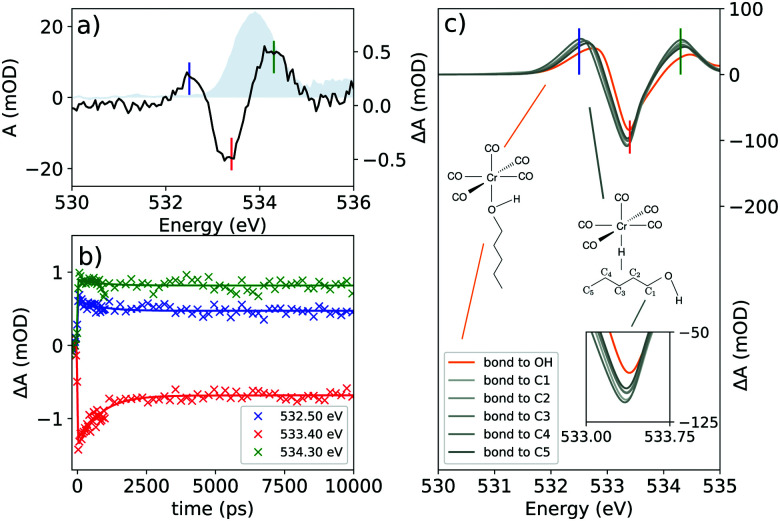
(a) The transient spectrum of Cr(CO)_6_ at the O K-edge at 100 ps delay. The static spectrum is shown in gray; (b) delay traces at selected features of the transient spectrum. The colors of the lines in (b) refer to the vertical lines drawn in (a); and (c) the theoretical transient spectrum at the O K-edge considering different binding sites for 1-pentanol.

**Fig. 5:**
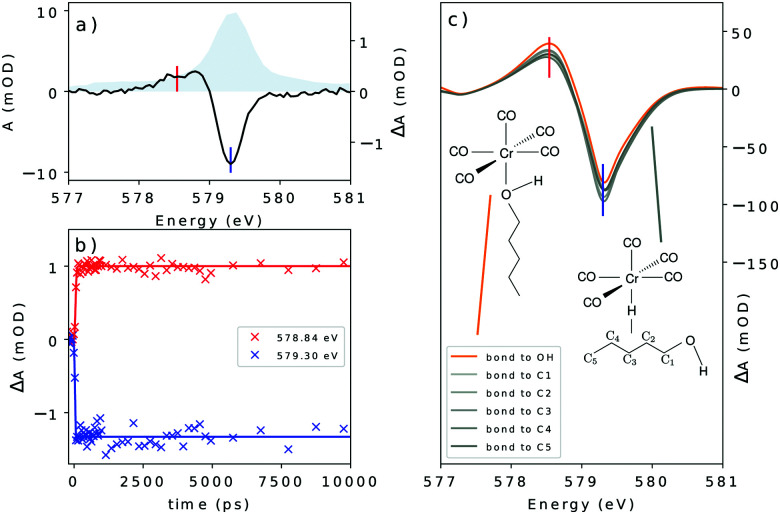
(a) The transient spectrum of Cr(CO)_6_ at the Cr L_3_-edge at 100 ps delay. The static spectrum is shown in gray; (b) delay traces at selected features of the transient spectrum. The colors of the lines in (b) refer to the vertical lines drawn at (a); and (c) the theoretical transient at the Cr L_3_-edge considering different binding sites for 1-pentanol.

The static data of both the chromium and oxygen are presented in Fig. 3. Time dependent data are presented in Fig. 4 and 5.

## 3 Computation

All theoretical computations were performed with the ORCA package.^21^ Structure optimizations were performed at the DFT level with B3LYP^22,23^ functional and def2-TVZP basis set with the def2/J auxiliary basis set^24^ in the RIJCOX approximation.^25^ Solvation effects were considered using the conductor-like polarizable continuum model (CPCM)^26^ for ethanol as an approximation. A list of the energy levels involved in the transitions of Cr(CO)_6_ is presented in Fig. 3b.

Spectral data were computed at the TD-DFT^27^ level with the same functional and basis set as that used for optimizations. The orbital excitation window was restricted to contain excitation from the 2p orbitals of Cr for the Cr L_3,2_-edge, and the 1s orbitals of the oxygen atoms for the O K-edge spectrum. Due to the self-interaction error and core–hole relaxation effects,^28–32^ the computed spectra were shifted by 12.2 eV at the Cr L_3,2_-edge and 14.3 eV at the O K-edge to match with the observed experimental features.

The computed spectra were broadened with a Voigt profile with Lorentzian FWHM of 0.2 eV for the Cr L_3,2_-edge and 0.16 eV for the O K-edge to account for the core–hole lifetimes of each edge. The Gaussian part of the Voigt profile has a FWHM of 0.8 eV.

## 4 Results

The X-ray absorption spectra (XAS) at the Cr L_3,2_- and O K-edges are presented in Fig. 3a. Below each XAS, the computation acquired with TD-DFT can be found. At the metal L_3_-edge, a peak centered at 579.30 eV is observed with a small shoulder centered at 577.81 eV. The L_2_-edge presents smaller intensity and spans from 585 to 590 eV centering at 587.68 eV with a shoulder at 586.34 eV. At the O K-edge, the XAS of Cr(CO)_6_ presents a single broad peak centered at 533.92 eV.

Furthermore, in Fig. 4 the time resolved data for the system at the O K-edge are presented. The transient spectrum in Fig. 4a was acquired with a delay of 100 ps after photoexcitation and presents a small absorption increase centered at 532.50 eV followed by a bleach centered at 533.40 eV and another absorption increase centered at 534.30 eV. Delay traces were measured for the three features and the results are presented in Fig. 4b. The solid lines were acquired by a numerical fit of the measured data with a single exponential yielding a decay constant of 790 ps convoluted with a 48.28 ps (FWHM) Gaussian function.

Time resolved data at the Cr L_3_-edge are presented in Fig. 5. The transient spectrum was acquired at a delay of 100 ps after photoexcitation. The spectrum presents a small absorption increase centered at 578.84 eV followed by a strong bleach feature centered at 579.30 eV. Delay traces measured at the maximum of each feature yielded step functions for both features in the transient spectrum. From the delay traces, a time-resolution of 62.32 ps (FWHM) was determined at the metal L_3_-edge.

## 5 Discussion

In the Cr(CO)_6_ complex, the field imposed by the strongest ligand in the spectroscopic series lifts the crystal field splitting raising the e_g_ set of the d orbitals above the π* orbitals from the ligand. The set of orbitals directly below the antibonding 6e_g_ set is the 3t_2g_ set with strong mixing of ligand π* and the metal d orbitals of t_2g_ symmetry. Virtual orbitals are shown in Fig. 3b. The established photochemical pathway of Cr(CO)_6_ after photoexcitation is depicted in Fig. 1.

In the O K-edge static spectrum (Fig. 3a), a broad peak centered at 533.92 eV is observed. This peak arises from the transition from the O 1s orbitals to the π* set of virtual orbitals (9t_1u_ and 2t_2u_, see Fig. 3b), from the ligand. The most interesting features, however, are seen in the time resolved data presented in Fig. 4.

**Fig. 6:**
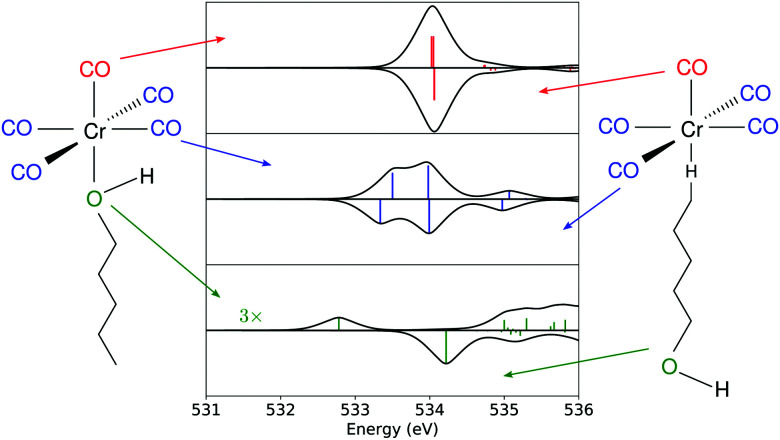
Computed spectrum at the O K-edge for each oxygen-bearing moiety in the substituted species. The upper part of each graph refers to the hydroxyl bound photoproduct, and the bottom part to the C_5_-alkyl bound product.

The transient spectrum shows three features. An absorption increase centered at 532.50 eV is attributed to the break in the symmetry of the CO centered levels, which unfolds into two peaks for Cr(CO)_5_(1-pentanol) in contrast with the ground state of Cr(CO)_6_. To disentangle the contributions in the transient spectrum, different models for the product must be considered. Looking at former studies, the coordination can take place through any of the carbon atoms of the solvent in weak σ interactions with any of the alkyl C–H moieties or through the hydroxyl end of the alcohol, which is the most stable product.^9,13^ As shown in Fig. 6, each of the three features can be traced back to the main contributions from one of the types of oxygen-bearing moieties in the photoproduct. By localizing the ligand core orbitals and restricting the orbital window in the computation for each of the oxygen centered orbitals, we can disentangle the individual contributions in the photoproducts and determine differences for alkyl or hydroxyl bound species. The axial ligand (red signal in Fig. 6) presents one peak due to the transition to the π* level of CO which is the same transition found for the carbonyls before photoexcitation.

This feature is slightly shifted to higher energies for alkyl coordination. The equatorial ligands (blue signal in Fig. 6) present a break in degeneracy of the ligand π* orbitals splitting the signal into two peaks. The splitting is slightly stronger when the solvent binds through the alkyl chain. The lower energy side of the transient signal with a small contribution from the incoming ligand (green signal in Fig. 6) brings the absorption increase centered at 532.50 eV. The computation shows a slightly higher broadening when the solvent binds through the alkyl chain. The depletion centered at 533.40 eV represents the difference between the main peak in Cr(CO)_5_(1-pentanol) and the ground state Cr(CO)_6_. In the photoproduct spectrum, the peak has contributions from both equatorial and axial CO ligands. The absorption increase centered at 534.30 eV is interpreted as the signature of the incoming solvent molecule as ligand.

**Fig. 7:**
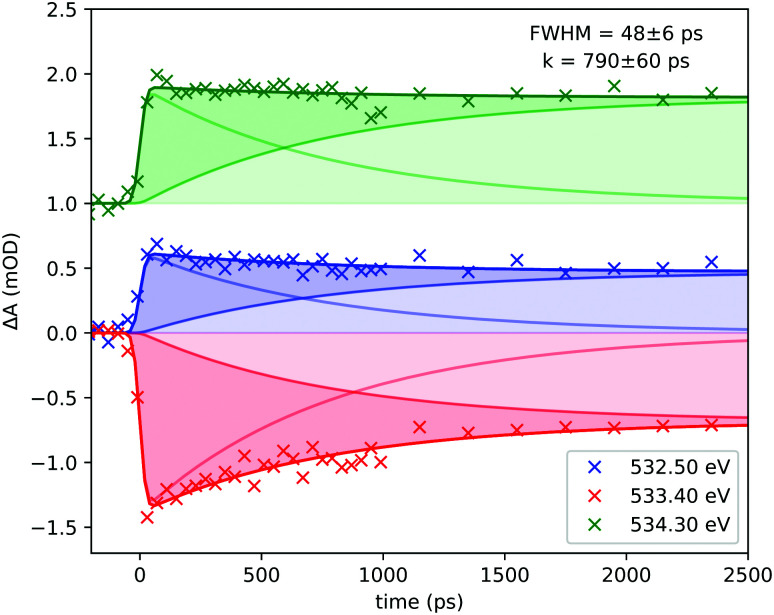
Detailed view of the components acquired in the fit of the experimental delay traces at the O K-edge. The data for the 534.30 eV curve were offset by 1 mOD for clarity.

From the delay traces presented in Fig. 4b, it can be seen that all features decay slightly on a sub-nanosecond timescale. From the computation shown in Fig. 4c it is noticeable in each feature that the hydroxyl bound complex presents a shift for higher energies. The models are compared in Fig. 4c. The fit of the data was performed numerically using a rate model consisting of short lived and long lived components. The fit was performed with the coupled experimental data. A table with the prefactors acquired is presented in the ESI.†Fig. 7 shows the components of each curve and the time constants acquired in the coupled model.

As discussed previously, the ground state of Cr(CO)_5_ is the singlet ^1^A_1_ in square pyramidal geometry.^4^ Comparing with other metal carbonyls, photo-chemical dynamics and reactions share common aspects, but also significant differences depending on the symmetry, state orthogonality and intermixing inherited from the saturated TM complex as well as the symmetry, electronic structure and intersystem crossings of the excited under-coordinated TM–CO complex fragments. In Fe(CO)_5_, *D*_3h_ symmetry is equivalent to strongly σ–π hybridized states, or expressed in a time picture as the extremely rapid MLCT-to-ligand field (LF) decay of 22 fs.^33^ In any case, the removal of one CO ligand brings the respective TM center into a formal 16 electron configuration, that allows low and high spin singlet and triplet configurations accompanied by different minimum energy structures causing vibrationally hot undercoordinated complex fragments. For the carbonyl complexes Fe(CO)_5_ and Cr(CO)_6_, ultraviolet light triggers photo-fragmentation by the ejection of a CO ligand, forming the unsaturated complexes Fe(CO)_4_^34^ and Cr(CO)_5_^6^ respectively. However, it is also apparent, that optical excitation into the “TM t_2g_–COπ*” derived MLCT state must differ for Cr(CO)_6_ in *O*_h_-symmetry or Fe(CO)_5_ in *D*_3h_-symmetry significantly: in Cr(CO)_6_*O*_h_ orthogonality preserves the orbital character, but allows with increasing TM–CO bond elongation crossing into the “TM e_g_–CO σ*” derived LF state. In the gas phase, sequential fragmentation ensues, whereas in solution, these under-saturated hot 16 electron systems are driven towards coordination with solvent molecules to re-establish the 18-electron closed shell (ligand exchange). Even though both Fe(CO)_5_^35,36^ and Cr(CO)_6_^3,4,37–39^ photo-eject multiple CO ligands in the gas phase and photosubstitute ligands in solution, their pathways differ. Gas phase Cr(CO)_6_ undergoes, after optical MLCT excitation and LF crossing, sequential ejection of multiple CO ligands.^3,4,37–39^ MLCT photoexcitation of octahedral Cr(CO)_6_ in solution reaches with increasing Cr–CO bond length the LF first excited singlet state from which the molecule dissociates. The Cr(CO)_5_ molecule decays to the singlet ^1^A_1_ ground state of square pyramidal geometry after going through the ^1^E′ singlet excited state of trigonal bipyramidal geometry^4^ (see Fig. 1). This unsaturated pentacoordinated Cr(CO)_5_ species in *C*_4v_ symmetry can finally be attacked by several types of strong^6,40,41^ or weak ligands^42,43^ maintaining in *C*_4v_ symmetry the local σ and π interaction to an incoming solvent molecule as it has been stabilized by efficient relaxation of vibrationally excited states.^6,44,45^ The dynamics of Fe(CO)_5_, however, is strongly determined by the mixed nature of Fe d and CO σ–π hybridized states in the ground, excited, fragmented and substituted states with a mixture of reactive singlet and triplet states^46,47^ while the *C*_4v_ Cr(CO)_6_ in the ^1^A_1_ ground state offers a single pathway for ligand attachment.

For the donor–acceptor interactions involved in the coordination seen in metal complexes, an electronic density is donated by the ligand to a metallic center. In the system studied here we considered that the 1-pentanol molecule has two kinds of donor centers – the alkyl and the hydroxyl. The hydroxyl center has a lone pair to be covalently shared between the metallic center and the ligand. The alkyl donor center, however, has no lone pair, and the electronic density donated to the metallic center can only be the one involved in the covalent bond between the C and H atoms. The interaction of three centers and two electrons between an alkyl moiety and a transition metal complex, C–H–M, was observed before and is termed an agostic interaction in the field of organometallics.^10^ Structures exhibiting a hydrogen bond of type OH⋯M as reported by Shubina *et al.*^48^ and found in other crystal structures^49–51^ should also be considered as possible intermediate configurations. However, the intermolecular interaction of the liquid phase alcohol, and the instability of the unsaturated metal carbonyl make a bond of this nature seem unlikely to be long-lived enough to affect the transient spectra here presented. We consider, therefore, that the lone pair would be the preferred coordination mode for the hydroxyl moiety.

With the photo-generated Cr(CO)_5_ stabilized in solution, the solvent molecules of the solvent cage point towards the complex through the alkane moiety as expected for a neutral molecule in an apolar solvent. Considering the former studies of Xie and Simon,^9^ Kotz *et al.*,^13^ and Shanoski *et al.*,^52^ a mixture of complexes of Cr(CO)_5_ is expected, in which, in the first steps after CO ejection, a majority of the formed product would present a mixture of the kinetically favorable alkyl bound complexes. The weak σ interaction of the C–H moiety exerts only a small perturbation of the π-derived states of the ligands. The bulky arrangement of the alkyl moieties might be a factor influencing their lability, with the weak interaction not being enough to hold the bond in place. Eventually, with the rearrangement, the unsaturated Cr(CO)_5_ would find the hydroxyl end of the solvent molecule and a more stable complex bound through the hydroxyl moiety would be formed. The σ interaction of the hydroxyl moiety together with the favourable steric arrangement holds the ligand and stabilizes the now 18 electron filled complex. The lability of the alkyl bond will cause the ligand to rearrange until the hydroxyl moiety is approached. The ability of the hydroxyl moiety to bind through σ interaction overcomes the ability in the alkyl moiety and it is expected that once the molecule reaches this structure it will remain stable at least on the nanosecond time-scale, which was probed in this experiment. The shift seen in Fig. 4c is interpreted as the source for the decay observed with the wavelength probed, shown in Fig. 4b. At the wavelengths probed, a blue shift of the spectral line causes an increase in absorption for the main peak in the case of bonding through the hydroxyl moiety, along with a decrease in the two neighboring features seen in the transient spectrum. However, for the alkyl bound products, no significant shift is seen between the considered species. These effects induce the decay of the transient absorption on subnanosecond timescales detected in the delay dependent intensities shown in Fig. 4. Due to the lability of the alkyl weak σ bond, it is expected that a long time after photoexcitation, only the hydroxyl-bound complex is visible in the spectrum. The rearrangement of long chain alcohols in d^6^ metal carbonyls was inferred by Shanoski *et al.* to take about 500 ps and 1.8 ns for 1-hexanol, measured by Kotz *et al.*^13^ Based on these past studies and on our computation, the decay in Fig. 4c is interpreted to arise from the ligand rearrangement and mainly reflects the change in the energy of the π* orbitals centered at the ligands. The fit of the delay traces in Fig. 4b yielded a time constant of 790 ps which lies in the range found for the rearrangement in previous studies.^13,52^

Looking at the Cr L_3_-edge, the static spectra present a curious signature. The rather structureless Cr L-edge XAS is in sharp contrast with that of the related isoelectronic species [Fe(CN)_6_]^4−^,^53^ in which the L_3_-edge is presented by two sharp peaks due to the transition to the ligand field e_g_ state, at the lower energy side, and due to the charge transfer transition at the higher energy side of the band. The spectral shape of Cr(CO)_6_, therefore, shows a stronger degree of back-bonding along with the strong field imposed by the CO ligand upon the metallic center which lifts the e_g_ levels derived from the d orbitals of the metal to energies higher than the MLCT level, as can be seen in Fig. 3a. In the Cr L_3_-edge mixed contributions of both sets of states to the main feature of the spectrum are observed.

The transient at the metal edge is dominated by a decrease in intensity. The depletion seen in Fig. 5 centered at 579.30 eV accompanied by an absorption increase centered at 578.84 eV is well reproduced by taking into consideration the expected substituted product. With the aid of calculations, the first peak is interpreted as arising from symmetry breaking which lifts the degeneracy of the 6e_g_ set of orbitals shifting the d_*z*^2^_ orbital to lower energies. Fig. 1 shows schematically the mechanism after photoexitation in which upon distortion of the octahedral geometry brought by the excited state, the d_*z*^2^_ orbital falls down rapidly in energy with the elongation of the Cr–CO bond distance and a dissociative potential is reached for any excitation below the LF set.

This behaviour is more associated with the elongation of the bond between the metal center and the ligand than to the coordination environment. Fig. 5c shows computations considering all the coordination sites in the solvent. There is no relative shift from one structure to another. This can be seen for both features probed. In the delay graph (Fig. 5c), both signals were fitted with a step function including a *σ* = 26 factor for the instrument response yielding a 62 ps FWHM for the time resolution of the experiment at this edge. Comparing the models, it can be inferred that the binding site of 1-pentanol does not have much influence on the metal centered orbitals. Therefore, the Cr L-edge has been shown to be insensitive to the coordination site of the solvent molecule in this spectral resolution. This behaviour is seen due to the different sets of orbitals being accessed by the different edges. The Cr L-edge transient spectrum of the substituted species, despite the coordination mode, will reflect changes to the states centered at the metal. The orbitals accessible by the metal edge are 3t_2g_ and 6e_g_ (see Fig. 3b) which interact weakly with the orbitals of 1-pentanol for any binding site as can be seen by the unchanged spectral shapes in Fig. 5c.

## 6 Conclusions

It has been shown for the photo-induced ligand substitution reaction of Cr(CO)_6_ in 1-pentanol that the substitution of the strong field ligand CO is followed by rearrangement of the incoming ligand. The σ interactions of varied strength provided by the incoming alcohol molecule break the degeneracy of the virtual set of π* orbitals centered at the equatorial ligands while almost no effect was observed at the remaining axial CO ligand. When considering the many sites of 1-pentanol, the alkyl ones were shown to be almost equivalent in their effects on the electronic structure of the complex with the hydroxyl site being the one that shifts the energy levels of Cr(CO)_5_ the most.

Through metal L-edge measurements, the degree of back-bonding was probed and it was compared with that of the isoelectronic [Fe(CN)_6_]^4−^. The high field imposed by the CO ligand lifts the e_g_ orbitals above the MLCT states imprinting the effect on the distinct spectral shape of the complex compared to other d^6^ analogous complexes. Due to the site selectivity of X-ray techniques, time resolved data gathered at multiple edges were crucial for tracking the reaction dynamics.

The photochemical reaction as seen at the O K-edge showed complexation of the solvent molecule to the unsaturated metal carbonyl as well as rearrangement of the incoming ligand to form the stable photoproduct Cr(CO)_5_(1-pentanol). Interpretation of the data guided by theoretical computations at the TD-DFT level confirms that the solvent molecule binds to the unsaturated metal carbonyl on a time scale below 50 ps and undergoes further rearrangement on a time scale of 790 ps to reach the final hydroxyl-bound photoproduct. Furthermore, it was shown that the effect of the incoming solvent on the energy levels of the carbonyl complex affects mostly the π*-derived states centered at the equatorial ligands, while the effect on the metal centered orbitals is minimal. The data gathered show the potential of X-ray absorption spectroscopy in investigating chemical processes through multiple perspectives.

## Author contributions

E. J. M.: data curation, formal analysis, investigation, and writing – original draft; M. F.: supervision, investigation, and data curation; R. B.: software and investigation; S. E.: data curation and investigation; V. V. C.: supervision, investigation, and formal analysis; A. F.: conceptualization, funding acquisition, and supervision; all authors contributed to writing – review and editing.

## Conflicts of interest

There are no conflicts to declare.

## Supplementary Material

CP-024-D1CP05834G-s001
