# Supplementary material for: Photo-induced ligand substitution of Cr(CO)6 in 1-pentanol probed by time resolved X-ray absorption spectroscopy
Source: Phys Chem Chem Phys. 2022 Jun 7;24(30):17979–85. doi: 10.1039/d1cp05834g (PMC9348521; doi:10.1039/d1cp05834g)
Supplement: CP-024-D1CP05834G-s001 [file CP-024-D1CP05834G-s001.pdf]

Electronic Supplementary Material (ESI) for Physical Chemistry Chemical Physics.
This journal is © the Owner Societies 2022

Supporting Information: Photo-induced Ligand Substitution of $Cr(CO)_6$ in 1-pentanol Probed by Time Resolved X-Ray Absorption Spectroscopy

Eric Johnn Mascarenhas,[1,2] Mattis Fondell,[1] Robby Büchner,[1,2] Sebastian Eckert,[1] Vinícius Vaz da Cruz,[1] and Alexander Föhlisch[1,2]

[1]*Helmholtz-Zentrum Berlin für Materialien und Energie GmbH, Institute for Methods and Instrumentation for Synchrotron Radiation Research, 12489 Berlin, Germany.*

[2]*Institute of Physics and Astronomy, University of Potsdam, Karl-Liebknecht-Str. 24-25, 14476 Potsdam, Germany*

COMPUTATION

All spectrum simulations were performed at the TD-DFT level with def2-TZVP basis set and def2/J auxiliary basis set. Three functionals were compared: PBE0[1], M06[2], and B3LYP[3, 4]. The comparison of the spectras for the Cr L-edge is presented in Fig. S1 and for the O K-edge in Fig. S2. Atomic positions in Angstroms used for computation of the x-ray absorption spectra are presented in tables S1-S8.

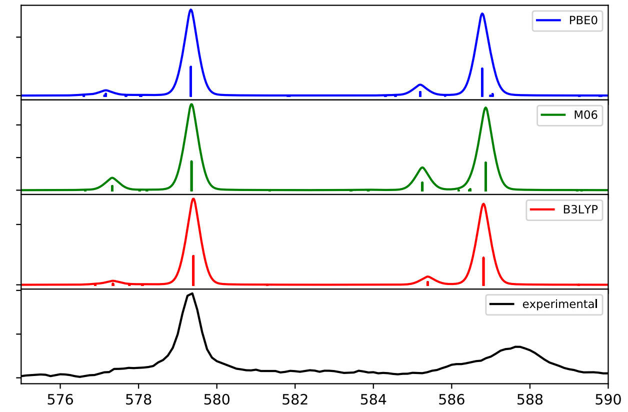

Figure S1 Comparison of the theoretical Cr L-edge spectra of $Cr(CO)_6$ computed with different functionals

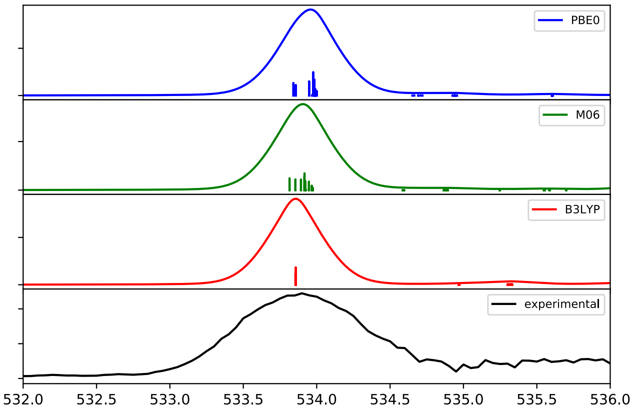

Figure S2 Comparison of the theoretical O K-edge spectra of $Cr(CO)_6$ computed with different functionals

Table S1 Atomic positions of $Cr(CO)_6$ in Angstroms optimized in DFT with B3LYP functional for computation of the O K, and Cr-$L_{3,2}$ edges x-ray absorption computations.

Atom	x($\mathring{A}$)	y($\mathring{A}$)	z($\mathring{A}$)
Cr	-0.00001	-0.00001	-0.00003
C	0.38831	-0.00194	1.88034
C	-0.38831	0.00192	-1.88039
O	0.61593	-0.00739	2.99773
O	-0.61591	0.00737	-2.99779
C	-0.91183	1.67679	0.18752
C	0.91181	-1.67681	-0.18758
O	-1.45069	2.67575	0.29759
O	1.45065	-2.67577	-0.29764
C	1.63887	0.93511	-0.34536
C	-1.63889	-0.93512	0.34531
O	2.61207	1.49221	-0.55251
O	-2.61209	-1.49221	0.55247

KINETIC MODEL DETAILS

Table S9 shows the fit parameters acquired for the delay traces. In the table, the first three energies displayed correspond to the peaks at the O K-edge. a_{sl} corresponds to the short lived coefficient acquired in the numerical fit, while a_{ll} corresponds to the long lived coefficient. The last two wavelengths correspond to the peaks seen in the Cr L_3-edge transient spectrum. The chromium L_3-edge delay traces were fitted by a step function where the coefficients considered where presented in the table in the column of the long lived component. Furthermore, k is the time constant of the decay of the functions at the O K-edge. σ is the width of the Gaussian used for the instrument response convolution and is related to the time resolution of the experiment by the Gaussian function relation FWHM $= 2\sqrt{2\ln 2} \sim 2.35482 \cdot \sigma$.

[1] C. Adamo and V. Barone, The Journal of Chemical Physics, 1999, **110**, 6158–6170.

[2] Y. Zhao and D. G. Truhlar, Theoretical Chemistry Accounts, 2008, **120**, 215–241.

[3] A. D. Becke, The Journal of Chemical Physics, 1993, **98**, 5648–5652.

[4] C. Lee, W. Yang and R. G. Parr, Physical Review B, 1988, **37**, 785–789.

Table S2 Atomic positions of $Cr(CO)_5(OHPent)$ in Angstroms optimized in DFT with B3LYP functional for computation of the O K, and Cr-$L_{3,2}$ edges x-ray absorption computations.

Atom	x($\mathring{A}$)	y($\mathring{A}$)	z($\mathring{A}$)
Cr	0.01115	0.02549	-0.17364
O	0.78575	-0.20910	1.87629
C	-0.59400	0.29253	-1.89704
O	-0.96701	0.45698	-2.97819
C	-1.10274	1.45161	0.46093
C	1.09541	-1.42878	-0.78560
O	-1.79340	2.29747	0.80112
O	1.71446	-2.29852	-1.19840
C	1.43016	1.26845	-0.51911
C	-1.44099	-1.18872	0.09523
O	2.24825	2.02216	-0.78720
O	-2.33674	-1.89719	0.17892
C	0.09771	-1.01999	2.86239
C	0.77636	-0.94052	4.21362
H	0.04746	-2.05125	2.50377
H	-0.91306	-0.61719	2.90477
C	0.04276	-1.77044	5.26674
H	0.82328	0.10582	4.52902
H	1.80822	-1.29684	4.11904
C	0.70847	-1.71222	6.64024
H	-0.01241	-2.81315	4.93584
H	-0.99054	-1.41672	5.35080
C	-0.03154	-2.53559	7.69173
H	0.76796	-0.66869	6.96601
H	1.74040	-2.06772	6.55404
H	-1.05791	-2.18018	7.81522
H	0.46242	-2.47603	8.66383
H	-0.07731	-3.58893	7.40293
H	1.71540	-0.47296	1.86511

Table S3 Atomic positions of $Cr(CO)_5$(PentOH) bound to C1 in Angstroms optimized in DFT with B3LYP functional for computation of the O K, and Cr-$L_{3,2}$ edges x-ray absorption computations.

Atom	x($\AA$)	y($\AA$)	z($\AA$)
Cr	-0.29712	0.35264	0.69301
C	0.76374	1.94706	0.68788
C	-1.36294	-1.24362	0.66332
C	-1.86691	1.40013	1.02347
C	1.27612	-0.68476	0.34444
C	-0.03294	0.17315	2.51261
O	1.40416	2.89313	0.72346
O	0.12873	0.06484	3.64728
O	-1.99248	-2.19655	0.68207
O	2.22052	-1.30477	0.17380
O	-2.79503	2.02331	1.26043
C	-0.53478	0.39540	-2.16000
H	-0.75196	0.76279	-1.11374
H	-0.04899	-0.57465	-2.12931
O	-1.78059	0.19532	-2.79092
H	-2.24744	1.04021	-2.84250
C	0.35771	1.44538	-2.79970
H	0.54378	1.15073	-3.83661
H	1.31995	1.41618	-2.27982
C	-0.21547	2.85906	-2.74210
H	-0.48922	3.09659	-1.70817
H	-1.13969	2.91489	-3.32718
C	0.75908	3.91516	-3.25810
H	1.03461	3.67654	-4.29031
H	1.68211	3.86298	-2.67178
C	0.18725	5.32890	-3.19120
H	0.90418	6.06612	-3.55840
H	-0.07215	5.59578	-2.16343
H	-0.71971	5.41311	-3.79554

Table S4 Atomic positions of $Cr(CO)_5$(PentOH) bound to C2 in Angstroms optimized in DFT with B3LYP functional for computation of the O K, and Cr-$L_{3,2}$ edges x-ray absorption computations.

Atom	x($\AA$)	y($\AA$)	z($\AA$)
Cr	-0.42939	0.91111	0.66147
C	-0.53768	2.80713	0.39101
C	-0.34224	-0.98459	0.91917
C	-2.34175	0.83850	0.78735
C	1.47893	0.99423	0.55417
C	-0.31600	1.17171	2.48346
O	-0.59728	3.94204	0.27795
O	-0.24117	1.33983	3.62078
O	-0.29209	-2.10697	1.13008
O	2.62103	1.05264	0.54632
O	-3.47597	0.80153	0.91987
C	-0.78082	0.42979	-2.13073
H	-1.51214	-0.29541	-1.77420
H	-0.22063	0.88323	-1.27675
C	-1.48801	1.57273	-2.84604
H	-0.77375	2.36187	-3.08343
H	-1.90219	1.19620	-3.78721
O	-2.51108	2.18873	-2.06421
H	-3.23110	1.55442	-1.95508
C	0.26832	-0.25822	-3.00094
H	1.01018	0.47977	-3.32192
H	-0.22648	-0.62106	-3.90769
C	0.97154	-1.42417	-2.31151
H	0.22371	-2.15604	-1.99173
H	1.46169	-1.06584	-1.40229
C	2.00596	-2.09950	-3.20832
H	2.77867	-1.38960	-3.51401
H	1.54015	-2.49562	-4.11421
H	2.49618	-2.92755	-2.69250

Table S5 Atomic positions of $Cr(CO)_5$(PentOH) bound to C3 in Angstroms optimized in DFT with B3LYP functional for computation of the O K, and Cr-$L_{3,2}$ edges x-ray absorption computations.

Atom	x($\AA$)	y($\AA$)	z($\AA$)
Cr	-0.33385	0.80167	0.74882
C	-0.29145	2.71035	0.58833
C	-0.38165	-1.10523	0.94626
C	-2.23380	0.84930	1.01319
C	1.56651	0.75563	0.51426
C	-0.08134	0.97146	2.56802
O	-0.25058	3.85175	0.55216
O	0.08133	1.08153	3.70249
O	-0.41217	-2.23200	1.13235
O	2.70648	0.74349	0.43412
O	-3.35381	0.87302	1.23572
C	-0.85696	0.40550	-2.07748
H	-1.59929	-0.30480	-1.71258
H	-0.32656	0.88143	-1.21320
C	0.20562	-0.31248	-2.90658
H	1.00540	0.38535	-3.16822
H	-0.26175	-0.63285	-3.84383
C	-1.54197	1.53467	-2.84670
H	-1.91019	1.10992	-3.78536
H	-0.79373	2.28478	-3.11738
C	-2.69992	2.19341	-2.10625
H	-2.36355	2.69642	-1.19873
H	-3.45262	1.45512	-1.82102
H	-3.18379	2.94227	-2.73598
C	0.80425	-1.53363	-2.22701
H	0.00987	-2.23183	-1.94048
H	1.33883	-1.24883	-1.32134
O	1.77449	-2.19340	-3.04714
H	1.32661	-2.50731	-3.84335

Table S6 Atomic positions of $Cr(CO)_5$(PentOH) bound to C4 in Angstroms optimized in DFT with B3LYP functional for computation of the O K, and Cr-$L_{3,2}$ edges x-ray absorption computations.

Atom	x($\AA$)	y($\AA$)	z($\AA$)
Cr	-0.75161	-0.50819	-0.60406
C	0.72013	0.42940	-1.39813
C	-2.24167	-1.43153	0.16741
C	-1.74613	-0.31109	-2.22795
C	0.21832	-0.64570	1.04482
O	1.60813	0.95300	-1.89077
O	-3.12097	-2.02104	0.59821
O	-2.33039	-0.22404	-3.20610
O	0.80973	-0.75123	2.01628
C	-1.74377	2.99924	-1.01035
H	-2.02066	4.03126	-0.78458
H	-2.42647	2.62780	-1.77663
H	-0.73424	3.00308	-1.42176
C	-1.83114	2.16174	0.25444
C	-3.22990	2.08305	0.85146
H	-2.73949	1.95497	2.94733
O	-4.64589	0.48649	3.95440
H	-5.55244	0.30847	4.23340
H	-1.53405	1.10917	0.03968
H	-1.10965	2.50225	0.99894
C	-3.27400	1.36484	2.19728
H	-3.60894	3.10329	0.96833
H	-3.89109	1.58459	0.13571
C	-4.69366	1.12515	2.67030
H	-2.76008	0.40228	2.12863
H	-5.22281	0.48923	1.95152
H	-5.23394	2.07626	2.74145
C	-0.06034	-2.11761	-1.18848
O	0.36837	-3.12187	-1.55392

Table S7 Atomic positions of $Cr(CO)_5$(PentOH) bound to C5 in Angstroms optimized in DFT with B3LYP functional for computation of the O K, and Cr-$L_{3,2}$ edges x-ray absorption computations.

Atom	x($\AA$)	y($\AA$)	z($\AA$)
Cr	-0.92754	-0.29363	-0.23242
C	0.18681	1.21478	-0.61080
C	-2.03621	-1.80976	0.14769
C	-2.19633	0.33692	-1.52508
C	0.33191	-0.91969	1.06686
O	0.87483	2.08722	-0.87542
O	-2.66896	-2.73844	0.34951
O	-2.93335	0.69726	-2.31886
O	1.10242	-1.32250	1.80758
C	-2.47848	1.01921	1.63161
H	-2.59765	2.10386	1.61868
H	-3.21491	0.60017	0.94992
H	-1.42785	0.87393	1.28763
C	-2.62738	0.43449	3.03043
C	-4.02367	0.67770	3.59872
H	-3.47517	0.58750	5.68431
O	-5.67779	-0.17737	6.87461
H	-6.56747	-0.01786	7.21197
H	-2.42821	-0.64043	3.00079
H	-1.87666	0.87354	3.69368
C	-4.19990	0.12121	5.00936
H	-4.22857	1.75383	3.60264
H	-4.76407	0.22386	2.93086
C	-5.59706	0.35799	5.54746
H	-3.99468	-0.95412	5.01151
H	-6.33552	-0.12739	4.89841
H	-5.81633	1.43224	5.55973
C	-0.04573	-1.25080	-1.53660
O	0.51162	-1.84443	-2.35020

Table S8 Atomic positions of free CO in Angstroms optimized in DFT with B3LYP functional for computation of the O K-edge x-ray absorption computations.

Atom	x($\AA$)	y($\AA$)	z($\AA$)
C	-3.62024	0.13173	0.00000
O	-2.49548	0.13173	0.00000

Table S9 Delay traces fit parameters. a_{sl} stands for the short lived prefactor. a_{ll} stands for the long lived prefactor.

Wavelength	a_{sl}	a_{ll}	k	σ
532.50	6.2e-04±2e-5	4.71e-04 ±1e-5	790±60	20±2
533.40	1.38e-3±2e-5	6.8e-04±1e-5		
534.30	9.0e-04±2e-5	8.2e-04±1e-5		
578.84	-	9.9e-04 ±1e-5	-	26±4
579.30	-	1.329e-3±1e-5	-	
